# Hepatic gene expression profiling reveals protective responses in Atlantic salmon vaccinated against furunculosis

**DOI:** 10.1186/1471-2164-10-503

**Published:** 2009-10-30

**Authors:** Stanko Škugor, Sven Martin Jørgensen, Bjarne Gjerde, Aleksei Krasnov

**Affiliations:** 1 Nofima Marin, P.O.Box 5010, Ås 1430, Norway; 2 Department of Animal and Aquaculture Sciences, Norwegian University of Life Sciences (UMB), P.O. Box 5003, Ås 1432, Norway

## Abstract

**Background:**

Furunculosis, a disease caused with gram negative bacteria *Aeromonas salmonicida *produces heavy losses in aquaculture. Vaccination against furunculosis reduces mortality of Atlantic salmon but fails to eradicate infection. Factors that determine high individual variation of vaccination efficiency remain unknown. We used gene expression analyses to search for the correlates of vaccine protection against furunculosis in Atlantic salmon.

**Results:**

Naïve and vaccinated fish were challenged by co-habitance. Fish with symptoms of furunculosis at the onset of mass mortality (LR - low resistance) and survivors (HR - high resistance) were sampled. Hepatic gene expression was analyzed with microarray (SFA2.0 - immunochip) and real-time qPCR. Comparison of LR and HR indicated changes associated with the protection and results obtained with naïve fish were used to find and filter the vaccine-independent responses. Genes involved in recruitment and migration of immune cells changed expression in both directions with greater magnitude in LR. Induction of the regulators of immune responses was either equal (NFkB) or greater (Jun) in LR. Expression levels of proteasome components and extracellular proteases were higher in LR while protease inhibitors were up-regulated in HR. Differences in chaperones and protein adaptors, scavengers of reactive oxygen species and genes for proteins of iron metabolism suggested cellular and oxidative stress in LR. Reduced levels of free iron and heme can be predicted in LR by gene expression profiles with no protection against pathogen. The level of complement regulation was greater in HR, which showed up-regulation of the components of membrane attack complex and the complement proteins that protect the host against the auto-immune damages. HR fish was also characterized with up-regulation of genes for proteins involved in the protection of extracellular matrix, lipid metabolism and clearance of endogenous and exogenous toxic compounds. A number of genes with marked expression difference between HR and LR can be considered as positive and negative correlates of vaccine protection against furunculosis.

**Conclusion:**

Efficiency of vaccination against furunculosis depends largely on the ability of host to neutralize the negative impacts of immune responses combined with efficient clearance and prevention of tissue damages.

## Background

Furunculosis caused by *Aeromonas salmonicida *spp *salmonicida *is a bacterial disease affecting salmonid species, including cultured and wild Atlantic salmon (*Salmo salar L*.) (reviewed in [1,2]), salmonids and other fish species [3-7]. Disease may have local and systemic, acute, subacute and chronic forms with diverse symptoms ranging from erratic swimming and slight darkening of skin, to haemorrhage on the abdominal walls, viscera and heart and ulcerative lesions [8]. Furunculosis is caused with non-mobile, aerobic gram negative bacillus *Aeromonas salmonicida*. This is an opportunistic pathogen with diverse strains that are characterized with different virulence. Furunculosis may cause heavy losses in salmon aquaculture due to mortality, decrease of growth rates, feed conversion and fish quality. Large-scale vaccination made it possible to reduce the incidence of disease and the use of antibiotics [9]. At present commercial vaccines against furunculosis are widely used and a number of experimental vaccines has been tried [10,11]. Vaccination decreases mortality of Atlantic salmon but fails to prevent it completely. Difficulties in the development of neutralizing vaccines are most likely accounted for by the high diversity of *A. salmonicida *strains and mechanisms of pathogenicity, which can be determined with various factors including type three secretion system, A layer protein, lipopolysaccharide, iron binding and outer membrane proteins, peptidases and toxins of different nature (reviewed in [2,11]). Knowledge of the virulence factors is far from complete. The limited success of protection against furunculosis is also related to high individual variation of responses to vaccination in Atlantic salmon [12].

Vaccination against furunculosis provides pathogen specific protection [2,9,13]. This indicates an important role of acquired immunity, which is however insufficient for the complete prevention of mortality. Outcomes of disease may depend substantially on the events, which take place after the recognition of pathogen. Activation of B and T cells and the complement system results in mass production of humoral factors that regulate recruitment of immune cells in blood and infected tissues. This results in the orchestration of anti-bacterial defense, including effector mechanisms, neutralization and clearance of exogenous and endogenous toxins, pathogens, damaged cells and their components. Defensive responses form a complex network, which may vary substantially among individuals. There are many possible scenarios with successful and deleterious outcomes. We used multiple gene expression profiling to outline the mechanisms that determine success of vaccine protection against furunculosis in Atlantic salmon and to search for the correlates of protection. Microarray analyses have been performed in fish vaccinated against different pathogens [14-17], however, to our knowledge, this study is the first attempt to search for the correlates of vaccine protection.

## Results

### Design of analyses

**Figure 1:**
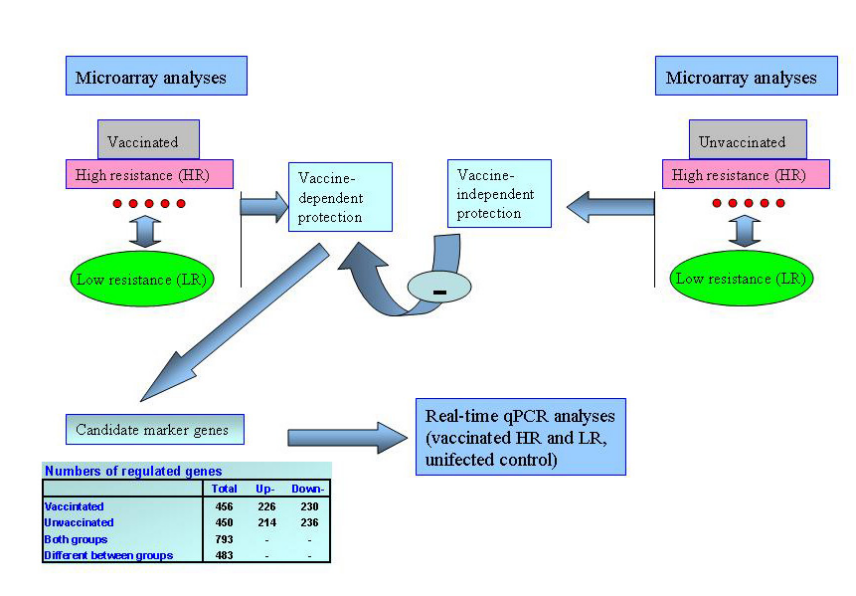
**Design of gene expression analyses**. Microarray comparison of HR (individual samples) with pooled LR outlined the gene expression changes associated with protection against *A. salmonicida*. Results obtained in naïve, unvaccinated fish indicated vaccine-independent protection and were used for the filtration of data obtained in vaccinated fish. Microarray analyses found genes with expression differences between LR and HR. The real-time qPCR analyses were conduced to verify the microarray results and to compare gene expression in the infected and uninfected fish. The numbers of differentially expressed genes are presented.

**Figure 2:**
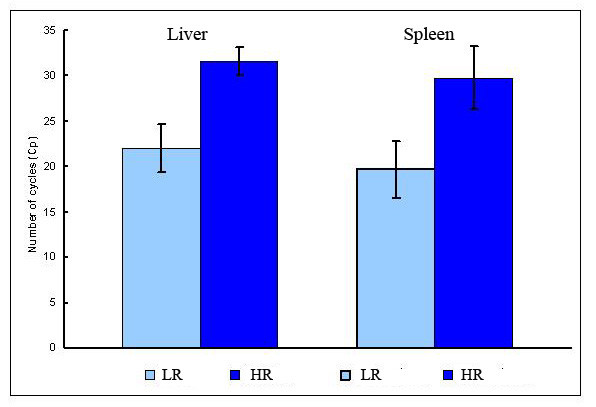
**Pathogen loads analysed by real-time qPCR in the liver and spleen of vaccinated salmon**. Data are cycle threshold (Ct) ± SD. Differences between LR and HR are significant (p < 0.001, ANOVA test, 8 fish per group).

To search for the correlates of vaccine protection against furunculosis, individual samples of fish that survived to the end of challenge test with no apparent symptoms of disease (high resistant, HR) were hybridized with pooled samples of salmon with manifestation of furunculosis (dark skin, lethargy, abnormal swimming behaviour and small haemorrhages at the base of fins) [8] at the onset of mass mortality (low resistance, LR). The same design of hybridization was applied to unvaccinated fish and the results were used to filter the vaccine-independent changes of gene expression (Figure 1). Pathogen was detected with qPCR in the liver and spleen of all analyzed fish though the load was substantially lower in HR (Figure 2). Pilot microarray analyses of different tissues (heart, spleen and liver of vaccinated fish) found greatest differences between HR and LR in the liver and this organ was chosen for the continuation of studies. Real-time qPCR results from all uninfected fish were used to form a calibrator sample in order to verify the microarray results and to determine the direction of gene expression changes. We report only genes that were associated with survival in vaccinated salmon.

### Recruitment of immune cells

**Figure 3:**
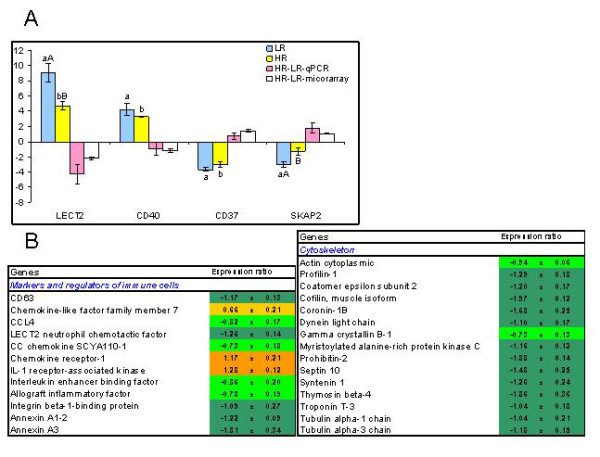
**Immune cell markers and genes involved in recruitment and migration of immune cells**. **A**: Real-time qPCR versus microarray analyses. Results demonstrated concordance in direction of change between the two techniques supporting differential regulation of the gene subset. Data for qPCR are ΔΔCt ± SE of 10 HR and 10 LR versus 8 fish in the unchallenged control and data for microarray are mean log_2_-ER ± SE of 6 HR fish hybridized to a pooled sample of 10 LR fish. Differences between HR and LR determined with microarrays and qPCR are shown. Different capital letters (A, B) denote a difference between challenged fish (HR and LR) while small letters (a, b) denote a difference between challenged and control fish (ANOVA, P < 0.05). LECT2 - leukocyte cell-derived chemotaxin 2, SKAP2 - Src kinase-associated phosphoprotein. **B**: Microarray results, examples of differentially expressed genes with significant differences between vaccinated and unvaccinated fish (t-test, n = 6 and 5, p < 0.05). Results for unvaccinated fish are not shown, data are log_2_-ER ± SE.

Up- and down-regulation of the immune cells markers (Figure 3) could be evidence for the changes in the composition of leukocytes in the liver. Mammalian homolog of CD37 (down-regulated) is expressed in mature B and T cells and myeloid cells [18] while CD40 (induced), also found in myeloid and B cells, is in addition expressed in fibroblasts, endothelial cells and in the basal epithelial cells [19]. Genes regulating cell motility also changed in both directions (up-regulated and down-regulated) with greater magnitude of responses seen in LR. Induction of leukocyte cell-derived chemotaxin (LECT2), which attracts neutrophils [20] was markedly greater in LR. Src kinase-associated phosphoprotein 55-related protein (SKAP2) was down-regulated. This protein is involved in signalling activated by interactions between cells and extracellular matrix (ECM) [21]. Microarray analyses revealed lower expression levels in HR of a large group of genes implicated in recruitment and motility of immune cells (Figure 3B). Both annexin A1 (ANXA1) and annexin A3 (ANXA3) showed noticeably lower levels in HR. The anti-inflammatory activity of annexins (ANXAs) is attributed mainly to their ability to interfere with neutrophil extravasation [22,23]. In mammals, ANXA1 is ubiquitously expressed, while ANXA3 has more selective expression patterns and tissue distribution [24]. Migration of cells is a highly regulated process controlled by cellular interactions with ECM. HR showed lower expression levels of integrin binding protein (ITGB1BP3/MIBP), which mediates reduced laminin cell adhesion and inhibition of matrix deposition [25]. Movement of cells involves the cytoskeleton. The 8-kDa dynein light chain (DLC8) is an essential component of the dynein motor complex that provides the driving force for microtubule-based transport within cells [26]. A potential link between cytoskeleton dynamics and gene regulation is implied by the fact that DLC8 binds to the inhibitor of the transcription factor NFkB preventing its translocation to the nucleus [27,28]. Profilin and cofilin that are involved in the restructuring of the actin filaments had lower expression in HR (Figure 3B). Same difference was observed in coronin 1-B, an actin-binding protein required for chemokine-mediated recruitment [29] and efficient cell protrusion and migration [30].

### Signal transduction and regulation of gene expression, anti-bacterial effectors

**Figure 4:**
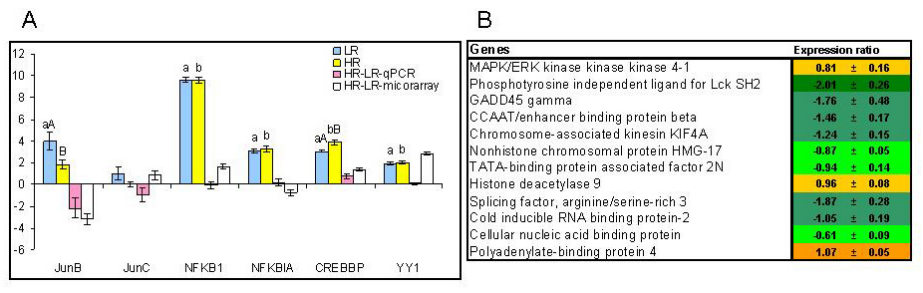
**Signal transducers and regulators of gene expression**. The figure shows expression changes indicative of recruitment and migration of immune cells. A. Real-time qPCR versus microarray analyses. Data for qPCR are ΔΔCt ± SE of 10 HR and 10 LR versus 8 fish in the unchallenged control and data for microarray are mean log_2_-ER ± SE of 6 HR fish hybridized to a pooled sample of 10 LR fish. Differences between HR and LR determined with microarrays and qPCR are shown. Different capital letters (A, B) denote a difference between challenged fish (HR and LR) while small letters (a, b) denote a difference between challenged and control fish (ANOVA, P < 0.05). NFKB1 - nuclear factor NF-kappa-B p105 subunit, NFKBIA - NF-kappa-B inhibitor alpha, CREBBP - CREB-binding protein, YY1 - YY1 transcription factor. B. Microarray results, examples of differentially expressed genes with significant differences between vaccinated and unvaccinated fish (t-test, n = 6 and 5, p < 0.05). Results for unvaccinated fish are not shown, data are log_2_-ER ± SE.

Six genes known for their key roles in closely intervened immune pathways were analyzed with both microarrays and qPCR (Figure 4A). All were up-regulated in the infected fish except for JunC in the HR group. The NFkB complex regulates numerous genes involved in the immune responses to bacteria [31]. Microarray analyses suggested greater expression level of NF-kappaB-p105 (NFkB1) in HR but qPCR analyses did not confirm difference between the study groups. Given a 1000-fold increase of NFkB1 in pathogen challenged fish in comparison with uninfected controls, discrepancies could be accounted for by the limited dynamic range of microarray analyses. Real-time qPCR also did not detect differences between the HR and LR in the expression of NF-kappaB inhibitor alpha (NFKBIA) that retains NFkB in the cytoplasm. Structurally and functionally related c-Jun, JunB and JunD together with the members of Fos and ATF/CREB protein families make up the transcriptional regulator AP-1, which is essential for the cooperative induction of many cytokine genes [32]. AP-1 mediated regulation is cell type specific, depends on the relative abundance of its subunits and presence of other nuclear factors. For example, the pleiotropic transcription factor Yin Yang 1 (YY1), whose higher levels were detected in HR by microarray, plays important roles in immune cells [33,34] and co-operates with AP-1 to regulate gene expression [35,36].

Microarray analyses (Figure 4B) showed differences between HR and LR in genes that regulate gene expression at different levels: signal transduction, promoter binding, modification of chromosomes, maturation and maintenance of mRNA. Several genes with known immune functions showed higher expression levels in LR, including the phosphotyrosine independent ligand for Lck SH2 or p62 that regulates activation of NFkB by TNFα [37]. General transcriptional activity seemed repressed in HR in comparison to LR as judged by the profile of TATA-binding protein associated factor 2N (TAF15), involved in the transcription complex assembly and transcription initiation by RNA polymerase II. Supportive of gene silencing in HR was the up-regulation of the histone deacetylase 9 [38]. On the other side, polyadenylate-binding protein 4 (PABPC4) that was markedly over expressed in HR is known for its ability to enhance the stability and translation of cytokine mRNAs [39].

**Figure 5:**
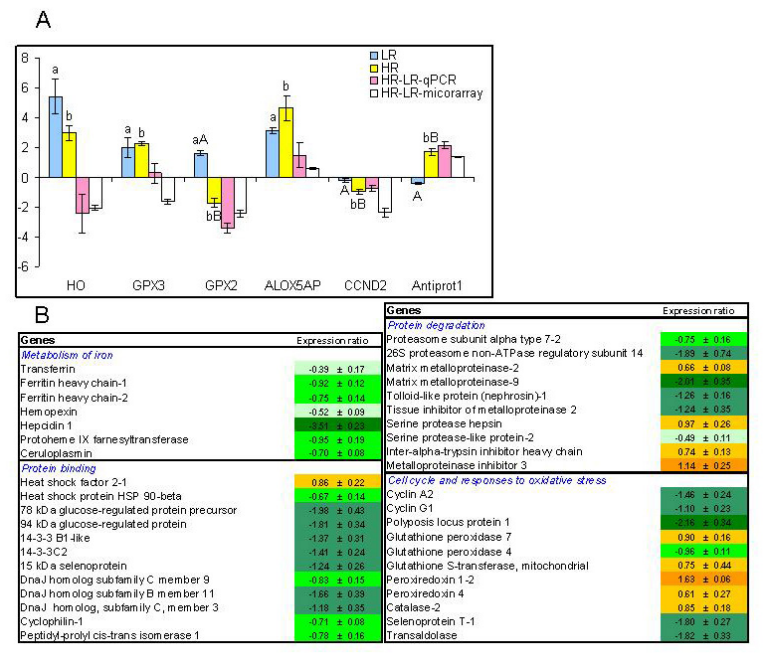
**Anti-bacterial effectors, markers of cellular and oxidative stress**. A. Real-time qPCR versus microarray analyses. Data for qPCR are ΔΔCt ± SE of 10 HR and 10 LR versus 8 fish in the unchallenged control and data for microarray are mean log_2_-ER ± SE of 6 HR fish hybridized to a pooled sample of 10 LR fish. Differences between HR and LR determined with microarrays and qPCR are shown. Different capital letters (A, B) denote a difference between challenged fish (HR and LR) while small letters (a, b) denote a difference between challenged and control fish (ANOVA, P < 0.05). HO - heme oxygenase; GPX3 - plasma glutathione peroxidase; GPX2 - gastrointestinal glutathione peroxidase; ALOX5AP arachidonate 5-lipoxygenase-activating protein; CCND2 - G1/S-specific cyclin D2; Antiprot1 - alpha-1-antiproteinase-like protein. B. Microarray results, examples of differentially expressed genes with significant differences between vaccinated and unvaccinated fish (t-test, n = 6 and 5, p < 0.05). Results for unvaccinated fish are not shown, data are log_2_-ER ± SE.

Up-regulation of NFkB and AP-1 by pathogens and cytokines induces mass production of immune mediators and effector proteins. Expression differences in proteosome components and extracellular proteases (nephrosin, matrix metalloproteinase 9 and tissue inhibitor of metalloproteinase) (Figure 5B) could be an evidence for the higher level of protein degradation in LR. On the contrary, improved resistance was associated with the activation of protease inhibitors that protect tissues from damage. Alpha-1-antiproteinase like protein (Antiprot1) was up-regulated in HR while LR showed no difference when compared to uninfected control (Figure 5A).

### Cellular and oxidative stress, metabolism of iron

In addition to cytokines and pathogens, NFkB and Jun proteins respond to various cell damaging factors, including free radicals and other genotoxic agents that can cause apoptosis, growth arrest, altered DNA repair or altered differentiation. AP-1 contains cysteine motifs that regulate its activity in response to oxidative stress [40]. NFkB can also activate protection against oxidative and cellular stress by providing anti-apoptotic and proliferation-promoting signals. A suite of chaperones and protein adaptors of different types (heat shock proteins, 14-3-3 proteins, glucose regulated proteins, DnaJ, cyclophilins) were expressed at higher level in LR fish (Figure 5B) and this could be evidence of cellular stress. Genes for proteins involved in regulation of redox status and protection against reactive oxygen species (ROS) also showed differences between the study groups. Five genes from this functional group that had higher expression levels in HR are presented in Figure 5B. In contrast, all genes involved in metabolism of iron had higher expression levels in LR. Transferrin is an extracellular transporter of iron and ferritin stores iron inside cells. Protoheme IX farnesyltransferase is an enzyme of heme biosynthesis while heme oxygenase (HO) plays a key part in heme degradation. Real-time qPCR showed induction of HO in all infected fish, consistent with its potent cytoprotective and anti-inflammatory functions [41], but the expression level was greater in LR (Figure 4A). Hemopexin transports heme to the liver for degradation and hepcidin regulates iron metabolism at different levels. Both genes respond to bacterial pathogens in various fish species [42-44]. Differences between LR and HR were also seen in regulators of cell cycle. Cyclin D showed lower level in HR. This gene links external cues with regulation of cell proliferation and directs entry to G1 phase by phosphorylation of retinoblastoma protein [45]. Our previous studies found marked activation of cyclin D with toxicity and pathogens [46,47].

### Complement

**Figure 6:**
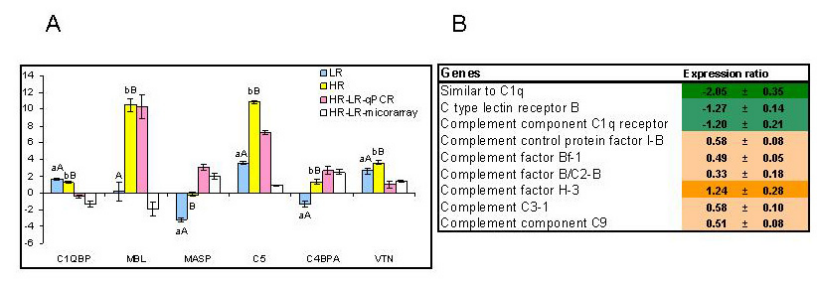
**Genes involved in the complement cascade**. A. Real-time qPCR versus microarray analyses. Data for qPCR are ΔΔCt ± SE of 10 HR and 10 LR versus 8 fish in the unchallenged control and data for microarray are mean log_2_-ER ± SE of 6 HR fish hybridized to a pooled sample of 10 LR fish. Differences between HR and LR determined with microarrays and qPCR are shown. Different capital letters (A, B) denote a difference between challenged fish (HR and LR) while small letters (a, b) denote a difference between challenged and control fish (ANOVA, P < 0.05). C1QBP - complement component 1, Q subcomponent binding protein; MBL - C-type mannose-binding lectin; MASP - mannan-binding lectin serine protease, C5 - complement component C5, C4BPA - complement component 4 binding protein, alpha, VTN - vitronectin. B. Microarray results, examples of differentially expressed genes with significant differences between vaccinated and unvaccinated fish (t-test, n = 6 and 5, p < 0.05). Results for unvaccinated fish are not shown, data are log_2_-ER ± SE.

**Figure 7:**
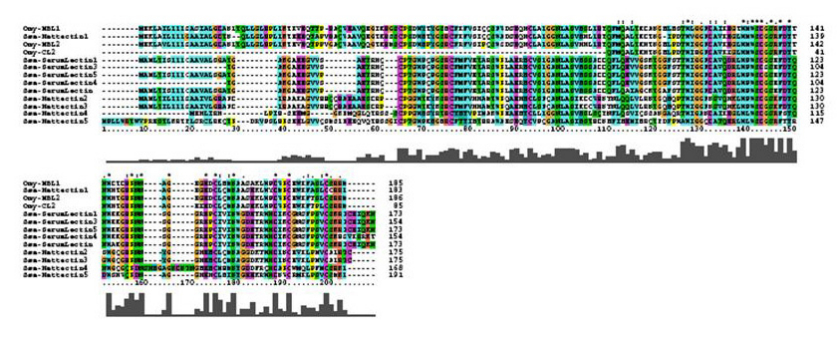
**Alignment of rainbow trout and Atlantic salmon lectins**. Sequence information includes the Genbank accession number; species is indicated as Omy for *Oncorhynchus mykiss *and Ssa for *Salmo salar*. Omy185132516 is encoded by the transcript spotted on the microarray. The sequences were aligned with ClustalX. D. Alignment of the predicted complement component 1, Q subcomponent binding protein (C1QBP) from three fish species with the human ortholog; species is indicated as Omy for *Oncorhynchus mykiss*, Ssa for *Salmo salar*, Dr for *Danio rerio *and Hs stands for *Homo sapiens*. The sequences are available at the accession numbers: human DQ891331, salmon paralog 1, salmon paralog 2, trout paralog 1 and trout paralog 2. The sequences were aligned with ClustalX.

The complement is a complex system consisting of 3 pathways (classical, alternative and lectin), which helps to kill and clear pathogens. The complement components are present in plasma as inactive proteins and the biochemical cascade is triggered by recognition of pathogens. In higher vertebrates, the complement pathways are activated in different ways. The C1Q component of C1 complex of the classical pathways (CP) binds to antigen-antibody complexes thus linking the innate and adaptive arms of immunity. C-type mannose-binding lectin (MBL) has the same role in lectin pathway (LP) but unlike C1Q commonly does not require antibody. The alternative pathway (AP) does not include specialized pathogen binding proteins. To date, the sensor proteins of CP have not been identified in salmonid fish. We included in analyses the C1Q binding protein (C1QBP), which showed greater expression in HR (Figure 6A). C1QBP is a ubiquitously expressed protein found intracellularly, on the cell surface, in plasma and the extracellular matrix. It interacts with a host of proteins including the globular heads of C1Q molecules, thus potentially modulating the numerous C1Q-mediated functions [48]. The functional analog of mammalian MBL is also unknown. Microarray results suggested down-regulation of a C type MBL in HR. However qPCR analyses found a 1000-fold induction of MBL in HR while there was no difference between LR and the uninfected control. This disagreement can be accounted for by the limited ability of cDNA microarrays to discriminate between transcripts of structurally similar members of multi-gene families. Blastx search of the MBL sequence presented on the microarray (Genbank CA376643) found three rainbow trout and nine Atlantic salmon proteins. Despite considerable sequence divergence (similarity ranged within 38-83%) the salmonid lectins contain highly conserved domains (Figure 7) that, hypothetically, may cross-hybridize. The actual number of salmonid MBL-like proteins and their relation to the complement system remain unknown. The genes for other serum components of the complement had greater expression levels in HR. These are serine proteases of lectin (MASP) and alternative pathways (factors B), C3, the convergence point of all complement pathways, C5 and C9, the parts of membrane attack complex. Factor H is a regulator of the alternative pathway while vitronectin (VTN) protects tissues against damages caused by the terminal membrane attack complex. The cell surface receptors of C1Q and C type lectin had higher expression in LR (Figure 6B). We show these genes since theoretically, they may interact with the complement system. However, their roles in salmonid fish are unknown and await exploration.

### Clearance and reparation

**Figure 8:**
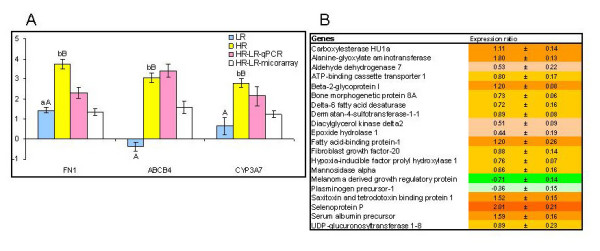
**Genes involved in tissue protection, clearance and reparation**. A. Real-time qPCR versus microarray analyses. Data for qPCR are ΔΔCt ± SE of 10 HR and 10 LR versus 8 fish in the unchallenged control and data for microarray are mean log-ER ± SE of 6 HR fish hybridized to a pooled sample of 10 LR fish. Differences between HR and LR determined with microarrays and qPCR are shown. Different capital letters (A, B) denote a difference between challenged fish (HR and LR) while small letters (a, b) denote a difference between challenged and control fish (ANOVA, P < 0.05). FN1 - fibronectin, ABCB4 - liver bile salt export pump. B. Microarray results, examples of differentially expressed genes with significant differences between vaccinated and unvaccinated fish (t-test, n = 6 and 5, p < 0.05). Results for unvaccinated fish are not shown, data are log_2_-ER ± SE.

HR was characterized with markedly higher expression levels of genes that protect tissues from damages, neutralize and remove toxic compounds and products of cell degradation (Figure 8A). Fibronectin (FN1) is an acute phase protein required for protection and reparation of ECM [49]. CYP3A7 can metabolise a number of endogenous and exogenous compounds [50,51] while liver bile salt export pump (ABCB4) mediates transmembrane movement of phosphatidylcholine and cholesterol from liver hepatocytes into bile [52]. MA analyses found a panel of genes involved in clearance, detoxification and reparation of tissues with higher expression levels in HR (Figure 8B). Removal of cholesterol seems to have high importance as evidenced by the profile of ATP-binding cassette transporter 1 (ABCA1), a cholesterol efflux pump in the cellular lipid removal pathway [53]. The intermediary metabolic enzyme alanine-glyoxylate aminotransferase (AGXT) is involved in the detoxification of glyoxylate, a product of amino acid metabolism and purine degradation in Atlantic salmon [54]. Previously we reported up-regulation of this gene in the liver of trout exposed to toxicity [46]. A number of genes with higher expression in HR have roles in the transport and modification of lipids. The exact metabolic function of the up-regulated transporter fatty acid-binding protein-3 (FABP3) in Atlantic salmon's liver remains unclear [55]. Diacylglycerol kinase delta 2 (DGKD) is involved in the conversion of diacylglycerol to produce phosphatidic acid. One more lipid-binding protein, beta-2 glycoprotein I, also known as apolipoprotein H is a precursor of anti-bacterial peptides [56]. Differences between the study groups were observed in several genes encoding growth factors. Melanoma-derived growth regulatory protein (MIA) (down-regulated in HR) mediates detachment of cells from ECM structures enhancing their migratory potential [57]. The up-regulated fibroblast growth factor-20 is a potent mitogen that induces DNA synthesis and cell proliferation [58].

## Discussion

Vaccines development is targeted at the complete neutralization of pathogens via binding to antibodies or TCR. However to date this aim has not been achieved for many diseases of Atlantic salmon including furunculosis. Vaccination reduces mortality but fails to provide a complete protection as confirmed in this study. Vaccinated HR fish that survived to the end of challenge test still showed relatively high levels of infection in the liver and spleen and induction of genes known for strong responses to bacterial pathogens. For example NFkB was induced 1000-fold with respect to the uninfected control. Markedly lower pathogen loads in HR suggested that survival of the vaccinated salmon was most likely determined with the ability to suppress and clear bacteria. Comparisons of gene expression within the groups of vaccinated and naïve fish found large scale differences between HR and LR. It is noteworthy to mention that analyses with vaccinated fish did not find significant role of genes involved in adaptive immune responses. This was in contrast with our studies of salmon challenges with virus (ISAV), which showed clear dependence between resistance evaluated by time of survival and activation of adaptive immunity [59]. Rapid stimulation of Igs was also seen in salmon challenged with the parasite, salmon louse [60]. Apparently, pathogen did not stimulate further activation of adaptive immunity in the vaccinated fish. Therefore one may assume that vaccine-dependent protection of salmon infected with *A. salmonicida *was determined mainly with the events that take place after recognition of the pathogen. Given high complexity and diversity of this network and limited level of knowledge, multiple gene expression profiling provides an efficient approach to search for the protective mechanisms.

The gene expression analyses were designed with focus on the mechanisms of vaccine-dependent protection against furunculosis. Genes with greater expression changes in HR can be regarded as candidate markers of protection while opposite regulation may indicate either pathology or unsuccessful defense. Overall, expression changes that can be interpreted as active anti-bacterial responses tended to be greater in LR. We produced an indirect evidence for larger regulation of immune cells recruitment in LR. More detailed study of the immune cells populations is complicated with shortage of cell-specific markers for salmon. Interaction of recruited and resident cells with pathogen components and cytokines activates signal transducers and other regulators of gene expression. We found increase of several genes known for their key roles in responses to pathogens and the magnitude was either greater in LR or equal in LR and HR (NFkB).

A panel of genes for proteins involved in metabolism of iron had higher expression in LR. These regulations indicate the need to reduce the levels of free inorganic iron and heme. HO was activated in all infected fish but the magnitude was greater in LR. HO is the rate-controlling enzyme of the degradation of heme into iron, carbon monoxide, and biliverdin, which is subsequently converted to antioxidant bilirubin [61]. Extracellular iron ions bind to transferrin, which delivers it into cells. Within cells iron is stored as complex with ferritin. Damage of erythrocytes is one of the symptoms of furunculosis. Heme and iron catalyze production of free radicals through Fenton's reaction thus increasing risk of oxidative stress. Shortage of iron may suppress proliferation of pathogenic bacteria and therefore sequestration of bioavailable iron and heme is regarded as anti-bacterial defense. In case of furunculosis this strategy is obviously unsuccessful.

The complement system was the only group of immune genes that showed strong association with survival and several genes presented in Figure 8A can be considered as candidate markers of vaccine protection against furunculosis. The complement system is the major link between the effector anti-bacterial mechanisms of adaptive and innate arms of immunity. The role of complement in antibody mediated defense against *A. salmonicida *was demonstrated in rainbow trout. Combination of specific IgM and complement accelerated ingestion of bacteria and ingestion-activated respiratory burst in phagocytes [62]. The complement pathways converge at the level of C3 convertase, which initiates the anti-bacterial effector mechanisms. C3 and the downstream genes, the parts of membrane attack complex had higher expression levels in HR, especially C5. Mobilization of the effector complement mechanisms correlated with survival, however it remained unknown, which of the complement pathways was responsible for these changes. Increase of C1QBP, the negative regulator of CP was greater in LR. This could be regarded as indirect evidence for higher activity of CP in HR, however, the expression difference between the study groups was relatively small. Furthermore, it remains unknown if CP provides the only or the major connection between the complement system and acquired immunity in salmonid fish. Homolog to mammalian C1Q was found in lampreys, primitive vertebrates that lack adaptive immunity [63]. The link between the classical pathway and antibodies appeared in the course of vertebrate evolution but the timing of this event remains undefined. C1Q is the member of a large multi-gene family with diverse functions [64]. Analysis of non-redundant Atlantic salmon mRNA sequences with blastx revealed 17 distinct C1Q related transcripts. To our knowledge, the antigen-antibody complex binding protein of salmonid CP has not been identified so far. In theory, interaction between the fish complement and acquired immunity molecules can be mediated by MP. MBL interacts with immunoglobulins in mammals [65] despite the principal role of CP in recognition of antibody antigen complexes. The qPCR analyses showed dramatic up-regulation of MBL in HR. Given the lack of changes in LR, this gene is one of the most promising markers of the vaccine-dependent protection. However taking into account the presence of multiple lectins with unknown roles in Atlantic salmon, induction of MBL does not necessarily mean activation of MP. Furthermore, MASP showed no increase in HR in comparison with uninfected control. Expression profiles of the complement factors Bf and B/C2-B suggested higher activity of AP in HR, however, differences between HR and LR were minor. Diversity of putative sensor proteins in salmonid fish and limited knowledge on their functions impede interpretation of the gene expression data.

Importantly, HR showed greater expression of factor H, the negative regulator of AP, and vitronectin (VTN), a protein that protects host tissues from the complement damages. Similarly, the transcript encoding alpha antiproteinase-like protein was up-regulated in HR relative to LR. Vaccine-dependent resistance to furunculosis was clearly associated with the abilities to prevent and repair damages from pathogen and immune responses and to neutralize and clear toxic compounds of endogenous and exogenous origin. The genes shown in Figure 8 can be divided in three functional groups - tissue reparation, clearance and xenobiotic metabolism - each represented with a gene whose markedly greater up-regulation in HR was confirmed with two methods. Elevated levels of fibronectin can be beneficial since FN1 is involved in reparation of tissues at the wound contraction stage of wound healing [66]. Due to its opsonic properties, fibronectin takes part in the removal of unwanted substances in the liver by phagocytic Kupffer cells [67]. CYP3A4 metabolizes a strikingly large number of xenobiotics including bacterial toxins and more than half of modern prescription drugs. This gene is down-regulated in inflammatory conditions by a range of cytokines and NFkB [68,69]. Hence individuals with strong innate immune responses may have a higher risk of bacterial intoxication. The role of ABCB4 in bacterial diseases has not been reported so far. One may speculate that the transporter protein that directs phospholipids from liver to bile can be important for clearance of remains of killed cells and bacteria. Transcriptomic comparison of salmon with high and low resistance to pathogen indicated pivotal importance of processes that deserve more attention in studies of fish diseases.

## Conclusion

Gene expression analyses revealed significant differences between vaccinated fish with high and low resistance to furunculosis. We did not find strong association between survival and most anti-bacterial responses though HR showed higher expression levels of several complement components. Results suggest that outcomes of vaccination depend largely on the ability of host to prevent the negative impacts of immune responses and to repair damages. This can be illustrated with the inductions of protease inhibitors, negative regulators of complement, genes involved in metabolism of lipids and xenobiotics and growth factors. Studies outlined a number of genes with positive and negative correlation with protection.

## Methods

### Challenge trials

The unvaccinated Atlantic salmon was from 279 full sib families (the offspring of 140 sires and 279 dams) while the vaccinated fish were a random sample of fish from 150 of the 279 families (the offspring of 87 sires and 150 dams). The families were produced by Salmo Breed AS in November 2006 and were reared in separate trays and tanks at Nofima Marin Sunndalsøra to a body size suitable for individually tagging with pit-tags in July (30 fish/family) and September (15 fish/family) 2007. The 30 fish/family were vaccinated intraperitoneally with a six component oil adjuvanted vaccine from PHARMAQ on 2 to 4 October 2007.

The pathogen challenge trials were approved by The National Animal Research Authority  according to the 'European Convention for the Protection of Vertebrate Animals used for Experimental and other Scientific Purposes' (EST 123).

Two groups of fish were infected with furunculosis at VESO Vikan, Norway in two separate tanks; the unvaccinated fish on 2 October 2007 at an average body weight of 30 g and the vaccinated fish on 22 November 2007 at an average body weight of 46 g. Infection was performed by cohabitation of 300 salmon (shedders) injected intraperitoneally with a virulent strain of *A. salmonicida *O2. The trial with unvaccinated fish was terminated after 20 days when cumulative mortality reached 76% while the trial with vaccinated fish, including 100 unvaccinated fish from the same families as controls was terminated after 60 days when cumulative mortality was 39% (vaccinated) and 72% (controls). In both trials liver, spleen and heart were sampled from 10 fish with symptoms of disease (darker colour, unusual swimming behaviour) at 10% cumulative mortality (14 and 22 days after challenge of respectively vaccinated and unvaccinated fish) and 10 fish at the end of trials. These groups were designated respectively as low resistant (LR) and high resistant (HR). Tissue samples were immediately dissected in 6 mm^3 ^pieces using RNase-free procedures and preserved in >1:10 v/v of RNALater (Ambion, Austin, TX, USA) at 4°C overnight following storage at -20°C. Presence of *A. salmonicida *infection was confirmed on a selection of fish at each stage by inoculating tryptic soy agar with swabs from posterior kidney and incubating at room temperature overnight. In addition, levels of bacteria in liver and spleen from both stages were assessed by PCR (see below).

### Detection of pathogen

DNA was extracted from the liver and spleen of vaccinated fish using the Qiagen DNeasy Blood and Tissue kit (Hilden, Germany) according to manufacturer's instructions. Equal amounts of isolated DNA (1.2 μg) were used as templates in 12 μl qPCR reaction volumes. DNA fragment (accession number X64214), reported to be highly specific for *Aeromonas salmonicida *(Hiney et al, 1992) was used to design primers. The cycling conditions were 95°C for 5 min (preincubation), 95°C for 5 sec, 60°C for 15 sec, 72°C for 15 sec (amplification); 95°C for 5 sec, 65°C for 1 min (melting curve); 55 cycles were performed.

### Microarray analyses

The salmonid fish microarray (SFA2, immunochip) includes 1800 unique clones printed each in six spot replicates. The complete composition of platform and sequences of genes are provided in submission to NCBI GEO Omnibus (GPL6154). Total RNA was extracted with TriZOL (Invitrogen) and purified with Pure Link (Invitrogen) according to the manufacturer's instructions. RNA was quantified using a NanoDrop 1000 spectrophotometer (Thermo Fisher Scientific, Wilmington, USA) and RNA integrity assessed using Bioanalyzer (Agilent 2100 Bioanalyzer, Agilent Technologies, Waldbronn, Germany) all samples giving RIN >8 and high purity without DNA contamination. The liver was chosen based on the results of pilot hybridizations with different tissues. Individual HR samples (6 vaccinated fish and 5 naïve fish) were hybridized to pooled LR samples (equal contribution from 6 fish) from the same groups. RNA (20 μg in each sample) was labelled with respectively Cy5-dUTP and Cy3-dUTP (Amersham Pharmacia). The fluorescent dyes were incorporated in cDNA using the SuperScript™ Indirect cDNA Labelling System (Invitrogen, CA, USA). The cDNA synthesis was performed at 46°C for 3 hours in a 20 μl reaction volume, following RNA degradation with 0.2 M NaOH at 37°C for 15 min and alkaline neutralization with 0.6 M Hepes. Labelled cDNA was purified with Microcon YM30 (Millipore). The slides were pretreated with 1% BSA fraction V, 5× SSC, 0.1% SDS (30 min at 50°C) and washed with 2 × SSC (3 min) and 0.2 × SSC (3 min) and hybridized overnight at 60°C in a cocktail containing 1.3 × Denhardt's, 3 × SSC 0.3% SDS, 0.67 μg/μl polyadenylate and 1.4 μg/μl yeast tRNA. After hybridization slides were washed at room temperature in 0.5 × SSC and 0.1% SDS (15 min), 0.5 × SSC and 0.01% SDS (15 min), and twice in 0.06 × SSC (2 and 1 min, respectively). Scanning was performed with GenePix4100A and images were processed with GenePix 6.0 (Molecular Devices). The spots were filtered by criterion *(I-B)/(S_I_+S_B_) ≥ 0.6*, where *I *and *B *are the mean signal and background intensities and *S*_*I*_, *S*_*B *_are the standard deviations. Low quality spots were excluded from analysis and genes presented with less than three high quality spots on a slide were discarded. After subtraction of median background from median signal intensities, the expression ratios (ER) were calculated. Lowess normalization was performed first for the whole slide and next for twelve rows and four columns per slide. The differentially expressed genes were selected using a two step procedure. First, technical accuracy was assessed by difference of log_2_-ER from zero in six spot replicates (Student's t-test, p < 0.01). The mean values were calculated and a single value per fish was used in subsequent analyses. Second, the genes with technically significant changes in at least half of vaccinated and naïve fish were selected and difference between these groups was assessed by biological replicates (t-test, p < 0.05). At total, 13 microarrays were used, one for the heart and spleen and 11 for the liver. Complete microarray results are provided in Additional file 1 and the data were submitted to NCBI GEO Omnibus (GSE18120).

### Quantitative real-time RT-PCR

Twenty five genes were selected for qPCR analyses taking into account their functional roles and the results of microarray analyses (Table 1). All genes were analyzed in vaccinated (experimental) and uninfected (control) fish. Total RNA was extracted and quality assessed as described in the section above. The cDNA synthesis was performed on 0.5 μg of DNAse-treated total RNA (according to manufacturer's protocol for routine DNAse treatment, Turbo DNA-*free*™ (Ambion, Austin, TX, USA) using TaqMan^® ^Gold Reverse Transcription kit (Applied Biosystems, CA, USA) and oligo dT primers, according to manufacturer's protocol. PCR primers were designed using Vector NTI (Invitrogen) and synthesized by Invitrogen. The amplicon lengths set to be between 50 and 200 bases were checked on 1.5% agarose gel. PCR efficiency was calculated from tenfold serial dilutions of cDNA for each primer pair in triplicates. Real-time PCR assays were conducted using 2× SYBR^® ^Green Master Mix (Roche) in an optimised 12 μl reaction, using 1:10 diluted cDNA, primer concentrations of 0.4-0.6 μM each. PCR was performed in duplicates in 96-well optical plates on Light Cycler 480 (Roche) under the following conditions: 95°C for 5 min (preincubation), 95°C for 5 sec, 60°C for 15 sec, 72°C for 15 sec (amplification), 95°C for 5 sec, 65°C for 1 min (melting curve). 45 cycles were performed. Relative expression of mRNA was evaluated by ΔΔCT. Three commonly used candidate reference genes (18s rRNA, eukaryotic translation initiation factor 3 subunit 6 and elongation factor EF-1a) were tested and 18s rRNA was selected as the reference gene for all samples by the stability criteria. Dependence of gene expression on infection, vaccination and resistance was analyzed with ANOVA (p < 0.05).

**Table 1:** Real-time qPCR analyses.

**Genbank accessions, target**	**Primer sequence from 5' to 3'**	**PCR efficiency**
CA366296, Src kinase associated phosphoprotein	F GAGGTGCTCCCAGAGGATGACA R CAGTCCCACAAGCCCTGGTAGT	2.0
CA349943, C-type mannose-binding lectin	F TCCATTGCACTGGGCGATGC R CACTGCTTCCACCTGAGCCTCA	1.667
CA361395, Leukocyte antigen CD37	F TGCTGAGACAAGCTTCTTCATGCC R CGACATCGTAGCACTTCCACCAAT	1.696
CA364370, CD40	F CTGTAAACTGCACCCATACTGCGAC R ATGGGCTGAGGCTTGTCTTGTTC	1.716
CU068239, Leukocyte cell-derived chemotaxin 2	F CTGTGTTGTCAGAGTGCGAGATGGT R TACACACAATGTCCAGGCCCTGA	1.899
CA369467, 5-lipoxygenase activating protein	F TCTGAGTCATGCTGTCCGTAGTGGT R CCTCCCTCTCTACCTTCGTTGCAAA	1.751
CA366162, Alpha-1-antiproteinase-like protein	F CCACAAGGCTGTGCTGAGCGTA R TGAGCATGATGGTGTCTGGGAGAG	1.702
CA366315, YY1 transcription factor	F AAAGAAGACGACGCGCCCAG R GGTGTGGAGATGCTTCCTCATCG	1.893
CF752495, Jun B	F CATCAGAAGTCGGCTCGCTGAA R GGTGTCGGTGTGGTAGTGATGACA	1.743
CA341859, NFkB1	F CAGCGTCCTACCAGGCTAAAGAGAT R GCTGTTCGATCCATCCGCACTAT	1.685
CA343143, NFkB inhibitor	F TGGTAACCTTGTGAAGGAGCTGGA R GCTCAGCATGTTCTGTGGCTTCAT	2.00
CA361415, Jun C	F CAGCATGACACTGAACCTGGCTGA R GCAAGTTTGAGGAGCTGCACATCC	2.00
CA345853, Plasma glutathione peroxidase precursor	F CCTTCCAGTACCTGGAGTTGAATGC R CTCATGATTGTCTCCTGGCTCCTGT	1.904
CF753103, Glutathione peroxidase-gastrointestinal	F TGTACCTCAAGGAGAAGCTGCCGT R ATTAAGGCCATGGGATCGTCGC	2.00
CA363120, Heme oxygenase	F TGGGTCTGACCTGGGTCCTCTCAT R GAGGGTGGTTTCAGCGTTGAGC	1.664
CA368533, Vitronectin	F AAGCCCTTCGACGCCTTCCT R CCTCTGATGCCCCACTTGTCGTAG	2.00
CA382259, Complement component 4 binding protein	F TGGTGGAGTATCAGTGTGACAGGCA R GGTGGATTTGGCTCAAACTGTCCT	2.00
CA364804, Complement component 5-2	F AGAACTCTTCCGAGTTGGCATGGT R AGTGATGCTGGGATCCATCTCTGA	1.952
CA366393, Mannan-binding lectin serine peptidase	F TCAGGTGCTGACGGAGAGGTCA R GCACTCTGAATCCCTCTGGTAGGAG	1.971
CA387557, Complement component 1Q binding	F CGGTCTCTCTGGATGATGAGCCATA R CCACATCCACACGACACAGGAGTA	2.00
CA376069, Cytochrome P450 3A27	F CCAACCTGCTGAACGGAATGAA R AGAACTCCTTCACTTCGATGGCCT	2.00
CA378743, Fibronectin	F GCATGTCTGAGACGGGCTTCAA R AGTCACATCGGAAGTGTCCACTGC	2.00
CA384134, G1/S-specific cyclin D2	F CATCAGACCACAGGAGTTGCTGGA R AAGTCATTTGGAGTGACAGCCGC	2.00
CA363965, Liver bile salt export pump	F TACGACACCAACGTAGGTTCCCAGG R GGATCTTAGGGTCGCGGATGATC	1.686
CA385588, CREB binding protein	F CTCCAGCCCAGGCCAACTCC R GGCCAGGCAGGTGAGCTCCT	2.00
X64214, *A. salmonicida *genomic DNA fragment	F GTTTACCACGTAATCTGAATTGTTCTTTTC R ATTGCTTATCGAGGCAGCCAAC	1.890
AJ427629, 18S rRNA	F GCCCTATCAACTTTCGATGGTAC R TTTGGATGTGGTAGCCGTTTCTC	2.00

## Competing interests

The authors declare that they have no competing interests.

## Authors' contributions

All authors contributed to the overall experimental design. BG was responsible for the challenge test. SŠ and SJ carried out the gene expression analyses whilst AK analyzed the results and produced the first manuscript draft. All authors read, contributed to, and approved the final manuscript.

## Supplementary Material

Additional file 1 **Complete results of microarray analyses**. The data provided represent the gene ratios in salmon with high and low resistance to furunculosis. The table includes log_2 _(Expression ratios) and p-values of differential expression (t test). Click here for file
